# Hyperosmotic Infusion and Oxidized Surfaces Are Essential for Biofilm Formation of *Staphylococcus capitis* From the Neonatal Intensive Care Unit

**DOI:** 10.3389/fmicb.2020.00920

**Published:** 2020-05-13

**Authors:** Yue Qu, Yali Li, David R. Cameron, Christopher D. Easton, Xuebo Zhu, Minli Zhu, Mario Salwiczek, Benjamin W. Muir, Helmut Thissen, Andrew Daley, John S. Forsythe, Anton Y. Peleg, Trevor Lithgow

**Affiliations:** ^1^The Neonatal Intensive Care Unit, The Second Affiliated Hospital and Yuying Children’s Hospital of Wenzhou Medical University, Wenzhou, China; ^2^Infection and Immunity Theme, Department of Microbiology, Biomedicine Discovery Institute, Monash University, Clayton, VIC, Australia; ^3^Department of Infectious Diseases, The Alfred Hospital and Central Clinical School, Monash University, Melbourne, VIC, Australia; ^4^The Commonwealth Scientific and Industrial Research Organisation (CSIRO) Manufacturing, Clayton, VIC, Australia; ^5^Department of Materials Science and Engineering, Monash Institute of Medical Engineering, Monash University, Clayton, VIC, Australia; ^6^Department of Microbiology, The Royal Children’s Hospital, Parkville, VIC, Australia; ^7^Department of Paediatrics, University of Melbourne, Parkville, VIC, Australia

**Keywords:** *Staphylococcus capitis*, biofilms, bloodstream infections, NICU, central venous catheters, surface chemistry, oxidized surfaces

## Abstract

*Staphylococcus capitis* is an opportunistic pathogen often implicated in bloodstream infections in the neonatal intensive care unit (NICU). This is assisted by its ability to form biofilms on indwelling central venous catheters (CVC), which are highly resistant to antibiotics and the immune system. We sought to understand the fundamentals of biofilm formation by *S. capitis* in the NICU, using seventeen clinical isolates including the endemic NRCS-A clone and assessing nine commercial and two modified polystyrene surfaces. *S. capitis* clinical isolates from the NICU initiated biofilm formation only in response to hyperosmotic conditions, followed by a developmental progression driven by *icaADBC* expression to establish mature biofilms, with polysaccharide being their major extracellular polymer substance (EPS) matrix component. Physicochemical features of the biomaterial surface, and in particular the level of the element oxygen present on the surface, significantly influenced biofilm development of *S. capitis*. A lack of highly oxidized carbon species on the surface prevented the immobilization of *S. capitis* EPS and the formation of mature biofilms. This information provides guidance in regard to the preparation of hyperosmolar total parenteral nutrition and the engineering of CVC surfaces that can minimize the risk of catheter-related bloodstream infections caused by *S. capitis* in the NICU.

## Introduction

Premature newborns given intensive care and prolonged hospitalization in the neonatal intensive care unit (NICU) are peculiarly prone to infections, due to their immature immune system and invasive medical procedures such as catheterization (Beck-Sague et al., 1994). Catheter-related bloodstream infections (CRBSI) are common and occur at a rate of 1–18 infections per 1,000 central line days (Blanchard et al., 2013; Yumani et al., 2013; Dubbink-Verheij et al., 2017). These infections are often associated with significant mortality and morbidity and contribute to high healthcare costs (Payne et al., 2004).

*Staphylococcus capitis* is an emerging opportunistic pathogen causing bloodstream infections in the NICU (Wang et al., 1999; Rasigade et al., 2012; Cui et al., 2013; Cheong et al., 2016). Neonatal patients infected with *S. capitis* often experience more severe morbidity relative to that of non-*S. capitis* coagulase-negative staphylococci (CoNS) (Ben Said et al., 2016). One specific clone of *S. capitis*, NRCS-A, was found to be highly endemic in the NICU and may have caused most *S. capitis* sepsis in neonatal patients worldwide (Butin et al., 2017a, b; Stenmark et al., 2019). In the NICU, *S. capitis* has been frequently isolated from central venous catheters (CVCs) explanted from CRBSI patients, supporting an important role of CVCs as the reservoir of invading bacteria (Ory et al., 2019). Past studies suggested that biofilm formation by *S. capitis* on medical devices such as CVCs correlates with its potential to establish infections (De Silva et al., 2002; Bradford et al., 2006). A direct link between neonatal sepsis caused by *S. capitis* and CVC implantation, however, has been questioned (Wang et al., 1999; Butin et al., 2019). Regardless, the importance of medical device surfaces as a host for bacterial colonization and biofilm formation has been well-established (Salwiczek et al., 2014). Direct contamination of implanted CVCs by bacteria from the NICU environment, patients’ skin or microbial translocation from the gut, throat or nostrils of patients, and subsequently bacterial adherence and biofilm formation are considered the key initiating steps of CRBSI (Hitzenbichler et al., 2017).

Antibiotic treatments of *S. capitis* infections in the NICU are often suboptimal due to their intrinsic and emerging resistance to many first-line antibiotics, and their capacity to form biofilms on implanted medical devices (Butin et al., 2015, 2019; Zhou et al., 2015). Biofilm formation is a self-defensive strategy of many microorganisms to survive adverse environments including long-term antibiotic exposure (Otto, 2008, 2013). For members of the genus *Staphylococcus*, including *Staphylococcus aureus*, *Staphylococcus epidermidis* and *S. capitis*, a characteristic feature of their biofilm formation is the inter-cellular adhesion associated with the production of polysaccharide intercellular adhesin (PIA), a linear β-glucosaminoglycan (Poly-β-1,6-GlcNAc) attached to the cell surface (Mack et al., 1994; Heilmann et al., 1996; Cui et al., 2013). The synthesis and secretion of PIA in staphylococci depends on the gene cluster *icaADBC*; disruption of *icaADBC* prevents cellular aggregation and biofilm formation (Gerke et al., 1998; Vermont et al., 1998; Frebourg et al., 2000; Galdbart et al., 2000; Arciola et al., 2001; Cui et al., 2013).

New antibiofilm strategies such as ethanol lock therapy and impregnating catheter surfaces with antibiotics have been recently examined in large-scale clinical trials targeting pediatric and neonatal patients (Wolf et al., 2018; Gilbert et al., 2019). Failure of these strategies in preventing CRBSI in young populations highlights an urgent need for more effective prophylaxis. This study sought to establish a comprehensive understanding of the interactions between *S. capitis* and biomaterials at the pathogen-medical device interface, and to provide valuable insights into more effective preventative strategies against CRBSI caused by *S. capitis* in the NICU.

## Materials and Methods

### Strains and Growth Conditions

Seventeen *S. capitis* clinical isolates (isolates 6, 8, 11, 17, 18, 19, 21, 25, 44, 52, 57, 61, 70, 76, 77, 80, 91) from blood cultures of infants at the Royal Women’s Hospital NICU (Parkville, Victoria, Australia) with confirmed bloodstream infections were used in this study for biofilm formation under different conditions. All isolates except isolates 44 and 77 belong to *S. capitis* subsp. *urealyticus*; isolates 44 and 77 belong to *S. capitis* subsp. *capitis* (Cui et al., 2013). These isolates typify a large collection of strains responsible for 55 episodes of sepsis in the NICU over the period 2000–2005 (Bradford et al., 2006; Cui et al., 2013). Eight isolates belonging to the unique NRCS-A clone (isolates 6, 8, 11, 17, 18, 19, 21, and 25), based on *smal* pulsed-field gel electrophoresis (PFGE) analysis were further selected to study interactions between *S. capitis* and biomaterials with different surface characteristics (D’mello et al., 2008; Butin et al., 2017b). Biofilm-positive *S. epidermidis* strain RP62A (ATCC 35984) and biofilm-negative *S. hominis* strain SP2 (ATCC 35982) were included as controls for biofilm production.

### Biofilm Cultivation and Imaging

Bacterial biofilms were cultured in 96-well microplates and quantitatively assessed using an established method (Deighton et al., 2001). Tryptic soya broth (TSB, Oxoid), TSB with 4% NaCl, TSB with 1% glucose, and TSB with 4% ethanol were selected as biofilm growth media representing different environmental cues encountered in the NICU. Nine commercially available polystyrene microplates with different surface characteristics were tested, including DNA-BIND (Corning), Carbo-BIND (Corning), Universal-BIND (Corning), Not Treated (Corning), CellBIND (Corning), Ultra-Low-Attachment (Corning), Immobilizer (Corning), Tissue Culture Polystyrene (TCPS) from NUNC, and TCPS from BD Falcon. Biofilms were also established on 96-well microplate cutouts (BD Falcon) using TSB with 4% NaCl and qualitatively examined with confocal laser scanning microscopy (CLSI) as described previously (Qu et al., 2009). CLSI images were obtained on a Leica SP5 microscope. SYTO-9 (3.35 μM, 15 min, 22°C) and Alexa Fluor 555 conjugated wheat germ agglutinin (WGA, 10 μg/mL, 1 h, 22°C) were used to stain the biofilms sequentially. All biofilm experiments for quantitative analysis were repeated at least three times in triplicate, and qualitative assays were repeated at least three times.

### Structural Analysis of *S. capitis* Biofilms

The composition of *S. capitis* biofilms was analyzed by treating mature biofilms with extracellular polymer substance (EPS) matrix-disrupting chemicals/enzymes respectively, including DNase I at 5 mg/mL, proteinase K at 100 μg/mL, and sodium metaperiodate (NaIO_4_) at 10 mM, for 2 h at 37°C with shaking (75 rpm) (Qin et al., 2007). Different buffers were used to prepare chemical/enzymatic solutions (5 mM MgCl_2_ and 5 mM CaCl_2_ for DNase I, 20 mM Tris-HCl and 5 mM CaCl for proteinase K, and 50 mM sodium acetate, pH = 4.5 for NaIO_4_). This was to ensure the maximum efficacy and stability of these reagents. Control wells were treated with buffer alone. The biofilm composition analysis assays were repeated at least three times in triplicate.

### qPCR Analysis

Quantitative real-time (qRT)-PCR was used to assess expression of *icaADBC* and *icaR* by *S. capitis* after growth in TSB or TSB with 4% NaCl at 37°C for 5.5 h (Chaffin et al., 2012). Primers were designed based upon the *ica* sequence of *S. capitis* isolate 6 (Genbank JF930147.1; Table 1). As an internal control, 16S rRNA levels were quantified. Relative gene expression was determined using the 2^–ΔΔC_T_^ method (Schmittgen and Livak, 2008). These experiments were repeated on three different occasions in technical triplicate.

**TABLE 1:** Oligonucleotide primers used in this study.

*icaR F*	CCATAGATATATTGGAGGGATCA
*icaR R*	GTCCAATTATCCAGTGCACC
*icaA F*	TATGAACCACGTGCCATGTG
*icaA R*	CTTCATGTCCACCTTGAGCC
*icaD F*	AGGGAGAGCTTATTCATTGCG
*icaD R*	CTCCACGTTAAGAGCGATACG
*icaB F*	GGATATGATCACGCAGCCTC
*icaB R*	GCAGACACATTAGACGCCTC
*icaC F*	CACGGTTCAAATGATAAGCGC
*icaC R*	GAAACCGCTAAGAAGACGACC
*16S rRNA F*^a^	AGCAACGCGAAGAACCTTAC
*16S rRNA R^b^*	CAACATCTCACGACACGAGC

*^a^F, forward. ^b^R, reverse.*

### Atomic Force Microscopy (AFM)

An Asylum Research MFP-3D atomic force microscope (Santa Barbara, CA) was used to assess the surface topography of the bottom of microwells in different polystyrene microplates. Tapping mode was used for imaging in air with ultrasharp silicon nitride tips (NSC15 non-contact silicon cantilevers, Mikro-Masch, Spain). The tips had a typical force constant of 40 N/m and a resonant frequency of 320 kHz. Typical scan settings involved the use of an applied piezo deflection voltage of 0.6–0.7 V at a scan rate of 0.8 Hz. All images were processed (1st order flattening algorithm) using Igor Pro software and arithmetic mean roughness (Ra) values were extracted. The experiments were repeated three times.

### X-Ray Photoelectron Spectroscopy (XPS) Analysis

XPS analysis was performed on the bottom of microwells originating from 96-well microplates using an AXIS HSi spectrometer (Kratos Analytical Ltd., United Kingdom) equipped with a monochromated Al-Kα X-ray source at a power of 144 W (12 mA, 12 kV). Instrument settings can be found elsewhere (Li et al., 2014). XPS analysis was performed using a method similar to that previously reported (Telford et al., 2012). Data processing was performed using CasaXPS processing software version 2.3.15 (Casa Software Ltd., Teignmouth, United Kingdom). Binding energies were referenced to the C 1s peak at 284.8 eV (aromatic hydrocarbon). These experiments were repeated on three different occasions in duplicate.

### Surface Modification of Untreated Polystyrene Microplates

Ozone treatment and diethylene glycol dimethyl ether plasma polymer (DGpp) deposition were carried out on untreated polystyrene surfaces (Corning Not Treated microplates) to provide an oxidized surface chemistry. For ozone treatment, the microplate was placed in a UV/Ozone ProCleaner^TM^ (BioForce) and exposed for 5 min. For DGpp deposition, plasma polymerization was carried out in a custom-built plasma reactor described previously (Li et al., 2014). The plasma deposition of films was performed using a frequency of 125 kHz, load power of 50 W and initial monomer pressure of 20 Pa with a treatment time of 120 s (final pressure 61 Pa).

### Immobilization of Biofilm EPS Matrix on Microplate Surfaces

EPS matrix was isolated from staphylococcal biofilms following the method described by Taff et al. (2012). Biofilm matrix supernatants (100 μL) were added into microwells in TCPS microplates (BD Falcon) and Not Treated microplates (Corning) and incubated for 3 or 20 h, at 37°C with gentle shaking (75 rpm). After washing with distilled water, the biofilm matrices immobilized on the surfaces were stained with Alexa Fluor 488 conjugated WGA (10 μg/mL, 1 h in the dark) before imaging with an Olympus IX81 fluorescence microscope. Three biological repeats were carried out for this experiment.

### Biofilm Formation and Surface Chemistry of Pediatric Central Venous Catheters

Pediatric double-lumen central venous catheters with blue FlexTip^®^ (CVCs, Arrow, Teleflex Medical, North Carolina, United States) were pre-treated with fetal bovine serum (Sigma-Aldrich, Sydney, Australia) overnight before biofilm growth or surface chemistry analysis. This was to mimic the pre-exposure of implanted CVCs to human serum in late-onset catheter related bloodstream infections. CVCs were washed twice with PBS and cut to sections of 5 mm for biofilm analysis or 15 mm for surface chemistry analysis. For qualitative biofilm analysis, catheter sections were cultivated with *S. capitis* isolate 6 in TSB with 4% NaCl at 1 × 10^7^ CFU/mL for 24 h, cut open in the middle, and prepared for scanning electron microscopy (Uwamahoro et al., 2012). For catheter surface chemistry analysis, all samples were suspended over the sample bar using a mask. To analyze the interior surface of the catheter, the double-lumen tube was cut open in the middle, and carefully spread flat. XPS analysis and data analysis were performed as described before. Untreated CVCs were also tested to make comparisons. Surface chemistry analysis of the CVC was repeated three times in duplicate, and biofilm scanning eletron microscopy was repeated three times.

### Statistical Analyses

To analyze differences in biofilm formation under different environmental conditions and on different surfaces, one-way ANOVA tests were performed with Minitab 19 for Windows using a significance level of 0.05 (*p-*value). Spearman correlation test was performed to determine the strength of a monotonic relationship between biofilm formation of *S. capitis* and biomaterial surface properties.

### Ethics Review and Approval

This study used strains obtained from RMIT University, Australia. Monash University did not require the study to be reviewed or approved by an ethics committee because these strains were provided to the research team in isolation from any references to individual patients. The Australian National Statement on Ethical Conduct in Human Research covers the use of human participants, their data, or tissues or body fluids. Using these microorganisms was not considered subject to ethical review by the Human Research Ethics Committee.

## Results

### Hyperosmotic Conditions Are Essential for *S. capitis* Biofilm Formation in the NICU

**FIGURE 1:**
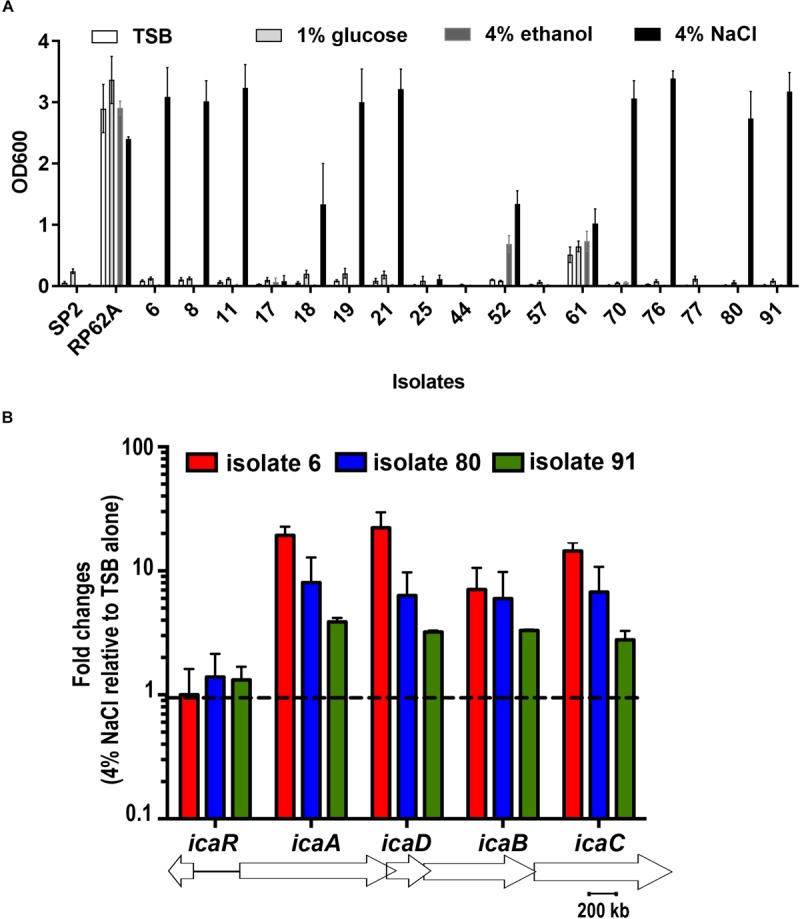
**(A)** Biofilm formation of 17 clinical *S. capitis* isolates on tissue culture polystyrene (TCPS) surfaces under different environmental conditions. Microplates were seeded with bacteria in the indicated media, including Tryptic soya broth (TSB) only, TSB + 1% glucose, TSB + 4% ethanol, and TSB + 4% NaCl. Biofilm formation was monitored using crystal violet staining. Error bars represent standard errors of the means (SEM). **(B)** The indicated isolates were grown in TSB or in TSB + 4% NaCl for 5.5 h and the expression of *icaADBC* and *icaR* at these two conditions were analyzed and compared by quantitative reverse transcription polymerase chain reaction (RT-PCR). Isolates 8, 11, 18, 19, and 21 were also examined and showed similar results as isolates 6. Error bars represent SEM.

BD falcon TCPS 96-well microplates were utilized for quantitative analysis of biofilm formation by 17 *S. capitis* isolates under different environmental conditions. All these 17 *S. capitis* isolates carry *ica* locus (Cui et al., 2013). Twelve out of 15 *S. capitis* subp. *urealyticus* clinical isolates formed biofilms. Among them, 10 isolates only produced biofilms in response to hyperosmotic conditions (4% (w/v) NaCl) but not under other environmental influences, such as TSB only, TSB with 1% glucose or 4% ethanol (Figure 1A). Two *S. capitis* subp. *capitis* (clinical isolates 44 and 77) failed to form biofilms under any of the studied conditions. As controls, *S. epidermidis* RP62A formed biofilms under all environmental conditions studied, while *S. hominis* SP2 did not form biofilms under any of the conditions (Figure 1A). Other hyperosmotic conditions such as the presence of NaCl at 2% (w/v), KCl or MgCl_2_ at 2–4% (w/v) were also able to induce biofilm formation of *S. capitis* (data not shown).

Quantitative RT-PCR showed that hyperosmotic treatment stimulated expression of *icaADBC* in representative clinical isolates of *S. capitis*, isolates 6, 80 and 91, with the mRNA levels for the *icaA, icaD, icaB, icaC* genes increased by 10–60-fold (Figure 1B).

### Structural Analysis of Biofilms Formed by *S. capitis* Under Hyperosmotic Conditions

**FIGURE 2:**
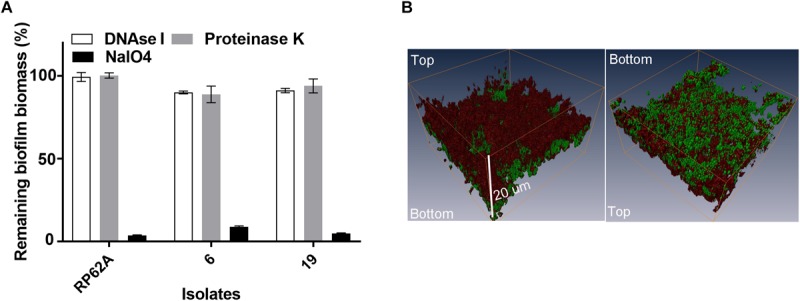
**(A)** Analysis of the matrix composition of mature biofilms formed by *S. capitis*. **(A)** Mature biofilms (20 h old) were treated with 5 mg/mL of deoxyribonuclease I (DNase I), 10 mM of sodium periodate (NaIO_4_), and 100 μg/mL of proteinase K for 2 h at 37°C, and the remaining biomass was assessed by crystal violet staining. **(B)** Confocal Laser Scanning Microscopy (CLSM) of *S. capitis* biofilms (isolate 6). Three-dimensional reconstructions show the staining pattern for polysaccharide intercellular adhesion [PIA, wheat germ agglutinin (WGA, red)] and cell mass (SYTO-9, green). The scale bar indicates the biofilm was ∼20 μm thick.

We quantitatively analyzed the composition of biofilms of isolates 6 and 19, by challenging established biofilms with component-disrupting chemicals or enzymes. Isolates 6 and 19 were selected as they represented two PFGE clusters after digestion with restriction enzyme *Sac*II (Cui et al., 2013). These two clusters were responsible for 34 out of 55 episodes of *S. capitis* bloodstream infections at the Royal Women’s Hospital NICU between 2000 and 2005 (Cui et al., 2013). The addition of sodium metaperiodate, not DNase I or proteinase K, to established biofilms caused detachment of ∼90% of the *S. capitis* biomass, suggesting a major role for PIA in the integrity of mature biofilms (Figure 2A). In order to visualize the relative mass of PIA in *S. capitis* biofilms, mature biofilms were formed on TCPS cutouts and stained with SYTO-9 and WGA. WGA is a lectin that specifically recognizes PIA in *S. epidermidis* biofilms (Sanford et al., 1995). Three-dimensional reconstruction of CLSM images revealed similar data from both isolates: the mass of PIA stained with WGA (Figure 2B, red) is equivalent to the cellular mass stained with SYTO-9 (Figure 2B, green) in biofilm structures and the PIA forms a surface cap across the top of the cellular layer.

### Features of Biomaterial Surfaces Dictate the Formation of Biofilms by *S. capitis*

**FIGURE 3:**
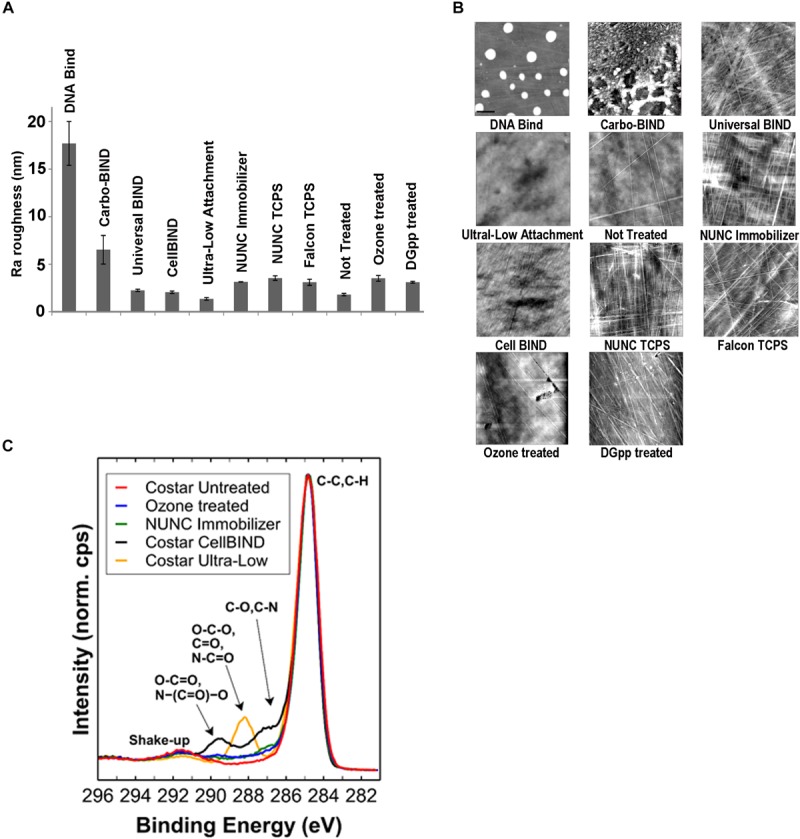
Physicochemical characterization of biomaterial surfaces. **(A)** Roughness (Ra) values of different surfaces used in this study calculated from atomic force microscopy (AFM) scanning. Error bars represent SEM. **(B)** AFM tapping mode images of surface topographies. Images were collected over 10 × 10 μm areas on microplates. For the DNA-Bind surface, the height trace image is displayed at 100 nm scale; all other images are displayed at 20 nm scale. **(C)** High-resolution C 1s spectral overlay including selected polystyrene samples. Spectra are normalized to maximum peak intensity of “Corning Not Treated” sample.

In the initial screening, no biofilm was formed by any of the 17 *S. capitis* isolates under any of the tested environmental conditions if TCPS was replaced by polystyrene microplates without surface treatment (Not Treated, Corning). This observation provided an opportunity to address the features of biomaterial surfaces that stimulates biofilm formation by *S. capitis* isolates from the NICU. We tested biofilm formation of eight *S. capitis* isolates of the NRCS-A clone in nine commercial microplates from different companies (see Methods). Only the CellBIND and TCPS microplates supported *S. capitis* biofilm formation (Table 2). Contact angle measurements reflect hydrophobicity of biomaterial surfaces, but these measurements only weakly correlate with biofilm formation of *S. capitis*, as found in this study [correlation coefficient *r* = −0.030, CI (−0.810, 0.469)]. AFM traces the topography of a surface and allows calculation of a mean surface roughness, but again only very week correlation was found between biofilm formation and surface roughness (Figure 3A) as measured by AFM (Figure 3B) [correlation coefficient *r* = 0.083, CI (−0.616, 0.709)]. XPS is a surface-sensitive technique used to quantify the elemental composition including oxygen, nitrogen and carbon content in the outer layer of biomaterials (Table 2). Here, Spearman’s correlation tests demonstrated a moderate correlation between biofilm formation and surface %O [correlation coefficient *r* = 0.567, CI (−0.216, 0.906)] or a strong correlation between biofilm formation and O/C ratio [correlation coefficient *r* = 0.611, CI (−0.160, 0.919)]. No such correlations were found for other elements (nitrogen or carbon species detected by XPS). Information regarding functional groups can be obtained from interpreting high-resolution spectra; as carbon is typically the most abundant element that can be detected by XPS in a polymer, it is often the most informative high-resolution spectrum (Figure 3C).

**TABLE 2:** Biofilm formation of *S. capitis* on different surfaces and their elemental composition [atomic% and atomic ratios (X/C)] derived from XPS survey spectra.

**96-well microplates**		Biofilm formation of isolates*									**% Composition**			**Atomic ratios**	
**Maker**	**Surface treatment**	**RP62A**	**6**	**8**	**11**	**18**	**19**	**21**	**17**	**25**	**O 1s**	**N 1s**	**C 1s**	**O/C**	**N/C**
Corning	DNA Bind	+	–	–	–	–	–	–	–	–	5.7 ± 0.3**	0.8 ± 0.1	93.5 ± 0.2	0.061 ± 0.003	0.008 ± 0.001
	Carbo-BIND	+	–	–	–	–	–	–	–	–	8.1 ± 1.6	4.7 ± 1.2	87.3 ± 2.7	0.093 ± 0.002	0.054 ± 0.015
	Universal-BIND	+	–	–	–	–	–	–	–	–	6.2 ± 0.5	NA	93.8 ± 0.5	0.066 ± 0.005	NA
	Not Treated	+	–	–	–	–	–	–	–	–	6.2 ± 0.3	NA	93.8 ± 0.3	0.066 ± 0.004	NA
	CellBIND	+	+	+	+	+	+	+	–	–	20.9 ± 0.5	1.2 ± 0.3	77.9 ± 0.6	0.269 ± 0.009	0.015 ± 0.004
	Ultra-Low Attachment	+	–	–	–	–	–	–	–	–	13.9 ± 1.0	11.0 ± 0.8	75.1 ± 1.7	0.185 ± 0.018	0.147 ± 0.014
NUNC	Immobilizer	+	–	–	–	–	–	–	–	–	12.2 ± 0.4	NA	87.8 ± 0.4	0.139 ± 0.005	NA
	TCPS	+	+	+	+	+	+	+	–	–	15.9 ± 0.2	NA	84.1 ± 0.2	0.190 ± 0.002	NA
Falcon	TCPS	+	+	+	+	+	+	+	–	–	16.4 ± 0.3	NA	83.6 ± 0.3	0.196 ± 0.004	NA
**MODIFIED SURFACES**															
Ozone treated		+	+	+	+	+	+	+	–	–	11.9 ± 0.4	NA	88.1 ± 0.4	0.135 ± 0.005	NA
DGpp treated		+	+	+	+	+	+	+	–	–	19.3 ± 0.2	NA	80.7 ± 0.2	0.239 ± 0.003	NA

*Only “+” and “—” are presented here to increase the readability of the table. +: biofilm-positive, with an OD600 > 0.24, –: biofilm-negative, with an OD600 < 0.12. Average biofilm biomass (OD600) of six biofilm-positive S. capitis strains on different surfaces were used for correlation analysis. **Presented in mean ± standard deviation. NA means there is no signal. XPS, X-ray photoelectron spectroscopy; O, oxygen; N, nitrogen; C, carbon; DGpp, diethylene glycol dimethyl ether plasma polymer.

### Highly Oxidized Surfaces Enable *S. capitis* EPS Immobilization and Promote Biofilm Maturation

**FIGURE 4:**
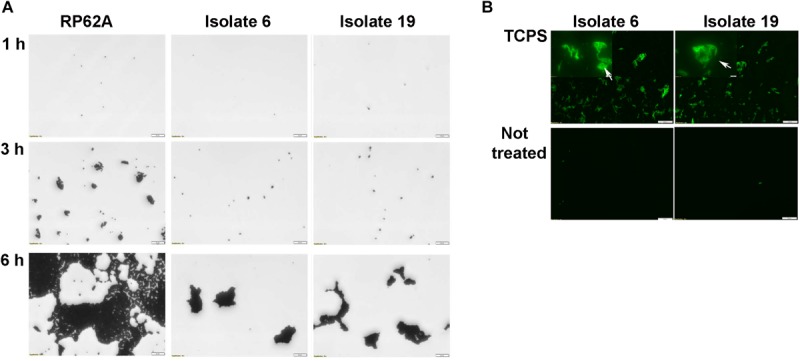
Characterization of biofilm development of *S. capitis* isolates 6 and 19 on tissue culture polystyrene (TCPS) and Not Treated surfaces. **(A)** Biofilm development on the Not Treat surface in the first 6 h monitored by crystal violet staining and light microscopy. *S. capitis* isolates 6 and 19 failed to network their microcolony structures and form biofilms on Not Treated surfaces. *S. epidermidis* RP62a, used as a control, formed typical macrocolonies on the Not Treated surface at 6 h. Scale bar = 20 μm. **(B)** Biofilm matrix of *S. capitis* isolates 6 and 9 was extracted, and the soluble extract was incubated with either TCPS or Not Treated surfaces, followed by wheat germ agglutinin (WGA) staining and fluorescence microscopy. Scale bar = 100 μm.

When biofilm formation was monitored in distinct untreated polystyrene microplates two representative strains of *S. capitis* (isolates 6 and 19) completed the early adherence phase of development, but failed to network the cell clusters even after 6 h incubation (Figure 4A). As polysaccharide was found to be the major component of *S. capitis* biofilm EPS matrix, a hypothesis was raised: a productive interaction between *S. capitis* EPS and highly oxidized surface might facilitate the networking needed for the subsequent phases of biofilm formation by *S. capitis*. Matrix materials extracted from mature biofilms of *S. capitis* could be immobilized on TCPS surfaces after a short exposure of 3 h, but not on untreated polystyrene surface even when the exposure was extended to 20 h (Figure 4B).

### Surface Chemistry of Pediatric Central Venous Catheters and *S. capitis* Biofilm Formation

**FIGURE 5:**
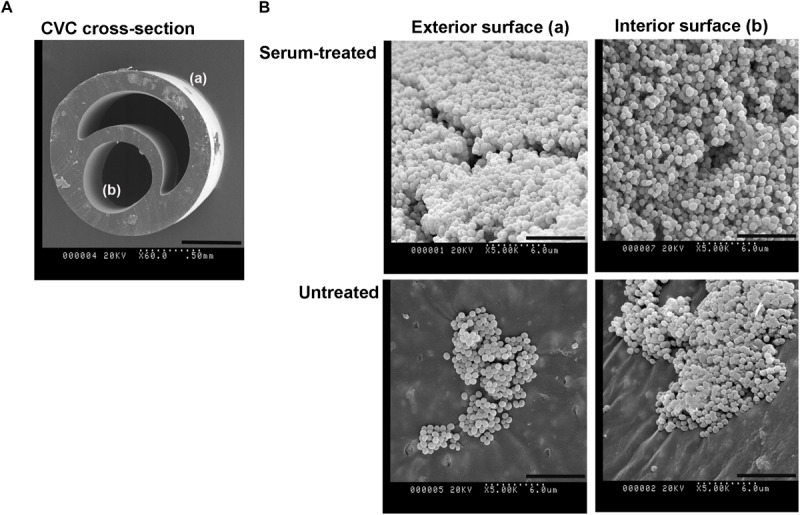
*S. capitis* biofilm growth on pediatric central venous catheters (CVCs). **(A)** A cross-section of the double-lumen central venous catheter is shown, denoting the exterior surface (a), and interior surfaces (b). **(B)** CVCs were pre-treated with or without fetal bovine serum (FBS) overnight. After cultivation with *S. capitis* isolate 6, catheter sections were prepared for scanning electron microscopy; representative micrographs show robust biofilms on surfaces of serum-treated CVCs that contain high percentages of oxygen element. Scale bar = 0.5 mm (cross sections images) or 6 μm (other images).

To determine whether the model polystyrene surfaces are reflective of the surface of vascular access devices, sections of pediatric CVCs were subjected to biofilm formation and XPS analysis (Figure 5 and Table 3). Surfaces of untreated CVCs were found to have typical polyurethane surface chemistry (as per manufacturer’s information), but additional properties also, and partially supported biofilm formation of *S. capitis* under hyperosmotic conditions (Table 3 and Figure 5). Both exterior and interior surfaces of the untreated CVC contain oxygen (10.7 ± 0.0% and 8.6 ± 0.2%, respectively) (Table 3). Exposure of CVCs to serum increased the oxygen level of exterior and interior surfaces to 16.9 ± 0.4% and 13.2 ± 0.8%, respectively, similar to that of biofilm-positive polystyrene surfaces, and supported more robust biofilm growth (Figure 5).

**TABLE 3:** Elemental composition [atomic% and atomic ratios (X/C)] of pediatric CVCs derived from XPS survey spectra.

**CVCs**	**Surfaces**	**% composition**							**Atomic ratios**	
		**Si 2p**	**S 2p**	**C 1s**	**N 1s**	**O 1s**	**Ba 3d**	**Na 1s**	**O/C**	**N/C**
Untreated	Exterior	5.2 ± 0.0*	NA	80.7 ± 0.2	3.5 ± 0.2	10.7 ± 0.0	NA	NA	0.132 ± 0.000	0.043 ± 0.003
	Interior	1.3 ± 0.1	0.2 ± 0.0	85.8 ± 0.3	4.0 ± 0.1	8.6 ± 0.2	0.2 ± 0.0	NA	0.100 ± 0.002	0.046 ± 0.001
Serum-treated	Exterior	9.4 ± 1.1*	NA	69.7 ± 1.1	3.8 ± 0.5	16.9 ± 0.4	NA	0.1 ± 0.0	0.243 ± 0.010	0.055 ± 0.006
	Interior	2.1 ± 0.0	NA	77.4 ± 1.4	6.9 ± 0.7	13.2 ± 0.8	NA	0.4 ± 0.0	0.170 ± 0.013	0.089 ± 0.010

**^∗^Presented in mean ± standard deviation. NA means there is no signal. CVC, central venous catheter; XPS, X-ray photoelectron spectroscopy; Si, silicon; S, sulfur; C, carbon, N, nitrogen; O, oxygen; Ba, Barium; Na, sodium.**

## Discussion

*S. capitis* has been only occasionally linked to several infections in adult patients, including infective endarteritis, prosthetic joint infections, catheter-related peritonitis, skin and soft-tissue infections, prosthetic valve endocarditis and meningitis (Oud, 2011; Takano et al., 2011; Basic-Jukic, 2017; Tevell et al., 2017; Lourtet-Hascoet et al., 2018; Natsis and Cohen, 2018; Yamamoto and Ohmagari, 2018). This opportunistic pathogen seems to have a greater clinical significance in the NICU, with a unique NRCS-A clone having caused many cases of severe sepsis in hospitalized neonates worldwide (Butin et al., 2016, 2017b). The current study examined biofilm formation of *S. capitis* isolated from the NICU and identified two key determinants that can be encountered in this specific clinical setting, including hyperosmotic conditions that activate the expression of biofilm operon *icaADBC* in *S. capitis* and highly oxidized surfaces that enable *S. capitis* EPS immobilization and biofilm maturation.

Biofilm formation of *S. capitis* has been found to be distinct from that of other coagulase-negative staphylococci that also cause CRBSI in the NICU (Cui et al., 2013). Using an evolutionary model proposed by Qin et al. (2007) and data from Cui et al. (2013), we determined that biofilm formation of *S. capitis* isolated from the NICU was still at an early-mid stage in evolution: the majority of isolates are biofilm-positive through their ability to secrete sufficient PIA (*ica*+, PIA+); several isolates are able to express *ica* gene in response to hyperosmotic stimuli, but fail to produce PIA or form biofilms (*ica*+, PIA−). This is not surprising as the NICU was first introduced in 1960s and the evolutionary driving forces arising from the selection pressure of antibiotics for *S. capitis* are relatively contemporary. The (*ica*+, PIA-) phenotype has been previously characterized: isolate 17, for example, has a small deletion that removes three hydrophobic amino acids from one of the critical transmembrane segments in IcaA, which would prevent IcaA assembly in the membrane and thereby prevent PIA production and biofilm formation (Cui et al., 2013). It is also possible that other *ica*-independent molecular mechanisms might have been involved. Carter et al. (2018) recently reported that in contrast to the majority of NICU-associated *S. capitis* isolates that harbor *embp*, 2 out of 29 neonatal *S. capitis* clinical isolates lack this gene. *Embp* encodes a multifunctional cell surface fibronectin binding protein and is critical for biofilm formation of *S. capitis* (Carter et al., 2018).

Unlike its close relative *S. epidermidis* that forms biofilms under many environmental conditions, *S. capitis* from the NICU appears to produce biofilms only under specific conditions. Most *S. capitis* isolates from the NICU require hyperosmotic condition for the activation of *icaADBC* and biofilm formation. Total parental nutrient (TPN) is frequently used in the NICU and might be the major source of hyperosmolarity. TPN has an osmolality up to 900 mOsm/L, ∼3 times that of physiological saline (Boullata et al., 2014). Previous studies have listed infusion of TPN as an independent risk factor for biofilm-related CRBSI in the NICU (Beck-Sague et al., 1994; Boullata et al., 2014). Other infusates that might confer hyperosmolarity in the NICU include 3% NaCl solution that are occasionally used for the management of hyponatremia. Our study has also found that an osmolarity as low as twice that of physiological saline readily induced biofilm formation of *S. capitis*. Preventing *S. capitis* infections by manipulating the osmolarity of infusates in the NICU is thus less feasible.

A more practical strategy to prevent CRBSI in the NICU is to use non-permissive biomaterials for CVCs. We focused on the interaction between *S. capitis* and biomaterial surfaces. Although silicone and polyurethane are often preferred biomaterials for implantable medical devices including CVCs, we used polystyrene microplates to establish screening conditions to understand the formation of biofilms by *S. capitis*. The uniform sized and flat surfaces of these polystyrene microplates provided an ideal platform for quantitative and physicochemical analysis of biofilm formation. The available knowledge of how these commercially available surfaces are fabricated from the manufactures allows us to comparatively analyze surfaces that have different biological responses.

Analysis of the surface hydrophobicity and topography did not reveal differences in the surfaces examined that would explain the success or failure of biofilm formation. These findings are consistent with studies by others that found no significant correlation between biofilm formation of *S. epidermidis* and *S. aureus* and the contact angle, free energy, or roughness of biomaterial surfaces (Tidwell et al., 1997; Gottenbos et al., 2001; Hook et al., 2012). Surface chemistry however seems to play a critical role in the biofilm formation of *Staphylococcus* spp. (Hook et al., 2012; Mikulskis et al., 2018). We show that oxygen species present in the surface layer are essential for biofilm formation of *S. capitis*: deliberate derivatization with oxygen species was necessary and sufficient to convert a surface that was non-permissive for biofilm formation into one which supported biofilm growth.

It has been proposed that surface cell growth correlates better to specific oxygen-containing functionalities than total oxygen content of a substratum (Tidwell et al., 1997; Hook et al., 2012). With the knowledge of how these surfaces are fabricated, and comparing O/C values presented in Table 2 and the high-resolution C 1s spectra (Figure 3C), it is expected that biofilm formation is also dependent on how the oxygen is presented at the surface, i.e., the functional groups. For example, surfaces of NUNC Immobilizer (*O*/*C* = 0.14) and Ozone treated (*O*/*C* = 0.14) microplates have almost identical concentrations of O, but biofilm formation occurs only in the Ozone treated microplate. A significant difference between these two microplates is the local environment of the O species on the surface which is influenced by how the oxygen was introduced to the surface. Ozone treatment of polystyrene surfaces is a non-specific modification that introduces a range of O species such as C-O, C = O, O-C-O, and O-C = O. This observation applies to all the surfaces that tested positive for biofilm formation in this study, as alternative discharge-based strategies employed herein such as Corona-gas treatment used for the CellBIND surface (as per manufacture’s specification) are non-specific. In contrast, biofilm-negative NUNC Immobilizer is described by the manufacturer as presenting an ethylene glycol spacer and a stable electrophilic group, i.e., the surface chemistry is well-defined. The surface of Corning Ultra-Low Attachment microplate, which presents one of the highest values for O/C (0.19) is another well-defined biofilm-negative surface, described as having a covalently bound hydrogel layer (as per manufacture’s specification), with the elemental quantification and C 1s spectrum suggesting a significant amount of amide groups present on the surface. All these suggest that surface treatments that introduce sufficient oxygen species in a non-specific manner result in biofilm formation. Findings derived from model polystyrene surfaces are further supported by experimental results from pediatric CVCs. Exposure of CVCs to serum significantly increased the oxygen composition of the surfaces, possibly due to the deposition of a conditioning film of host serum components, including proteins and electrolytes on the CVC surfaces. Such a conditioning film facilitates biofilm formation of *S. capitis*.

To address the detailed role of surface oxygen species in the biofilm formation of *S. capitis*, we further assessed the interaction between *S. capitis* biofilm EPS and biomaterial surfaces. It is well-known that EPS matrix plays an important role in the stability and antimicrobial resistance of mature biofilms (Flemming and Wingender, 2010). By biochemically dissecting the composition of *S. capitis* biofilms, we found PIA to be the predominant component of EPS matrix. This was further confirmed by high-resolution imaging using CLSM and PIA-specific staining. A specific binding between oxygen species and PIA was found: only on highly oxidized surfaces does PIA function as a crucial surface-binding adhesin, networking microcolonies and enabling the maturation stages of biofilm formation; otherwise similar surfaces were non-permissive to biofilm development.

Given the importance of biofilm formation in hospital-acquired infections, a deep understanding of the role that surface chemistry plays in this microbial developmental process offers opportunities for novel interventions. Based on our findings, we conclude that reducing the level of oxygen species on the biomaterial surface, for examples, using low-fouling surfaces for CVCs against protein adsorption (Salwiczek et al., 2014), as well as avoiding hyperosmotic treatments wherever possible would be a strategy to minimize infusion- and catheter-related *S. capitis* infections in the NICU. An evident limitation of this study is that it still suffers from an inability to comprehensively reflect the complexity of *in vivo* conditions in the NICU. Future studies that closely mimic the NICU environment and use CVCs with different surface oxygen levels should be conducted to verify the *in vitro* findings from this study.

## Data Availability Statement

The datasets generated for this study are available on request to the corresponding author.

## Author Contributions

YQ, TL, YL, and AP conceived and designed the experiments. YQ, YL, DC, and CE performed the experiments. YQ, TL, XZ, MZ, YL, DC, CE, HT, MS, HT, AD, JF, and BM analyzed the data. TL, HT, AD, JF, BM, AP, and YQ contributed reagents, materials, and analysis tools. YQ, TL, YL, and CE wrote the manuscript.

## Conflict of Interest

The authors declare that the research was conducted in the absence of any commercial or financial relationships that could be construed as a potential conflict of interest.
